# A New Look at an Old Disease: Recent Insights into the Global Epidemiology of Dengue

**DOI:** 10.1007/s40471-017-0095-y

**Published:** 2017-01-14

**Authors:** Tyler M. Sharp, Kay M. Tomashek, Jennifer S. Read, Harold S. Margolis, Stephen H. Waterman

**Affiliations:** Dengue Branch, Division of Vector-Borne Diseases, Centers for Disease Control and Prevention, 1324 Calle Cañada, San Juan, PR 00920-3860 USA

**Keywords:** Dengue, Epidemiology, Burden

## Abstract

**Purpose of Review:**

By all measures, the morbidity and mortality due to dengue are continuing to worsen worldwide. Although both early and recent studies have demonstrated regional differences in how dengue affects local populations, these findings were to varying extents related to disparate surveillance approaches.

**Recent Findings:**

Recent studies have broadened the recognized spectrum of disease resulting from DENV infection, particularly in adults, and have also demonstrated new mechanisms of DENV spread both within and between populations. New results regarding the frequency and duration of homo- and heterotypic anti-DENV antibodies have provided important insights relevant to vaccine design and implementation.

**Summary:**

These observations and findings as well as difficulties in comparing the epidemiology of dengue within and between regions of the world underscore the need for population-based dengue surveillance worldwide. Enhanced surveillance should be implemented to complement passive surveillance in countries in the tropics to establish baseline data in order to define affected populations and evaluate the impact of dengue vaccines and novel vector control interventions.

## Introduction

As is true for many emerging infectious diseases, increasing global circulation of dengue is a result of the expansion and urbanization of society [1]. The four genetically and antigenically distinct dengue virus (DENV) types [2•] diverged from a common ancestor roughly 1000 years ago [3]. Each virus independently shifted from a mosquito-monkey-mosquito cycle of transmission to becoming endemic in humans several hundred years ago [3, 4]. The advent of maritime transportation enabled the dispersal of *Aedes* mosquitoes and DENV-infected humans throughout the tropics, and dengue outbreaks were increasingly reported during the seventeenth century [3]. World War II played a prominent role in enabling co-circulation of multiple DENV types and subsequent epidemics of dengue hemorrhagic fever (DHF) in Southeast Asia in the 1950s [5]. *Aedes* mosquitoes were nearly eradicated from the Americas in the middle of the twentieth century [6]. However, unsustainable vector control strategies [7] allowed the resurgence of *Aedes* mosquito populations in the Americas, and epidemics of DHF were first reported throughout the region in the 1980s [8]. Thus, the global spread and increasing severity of dengue were enabled by increased population growth, more frequent and expansive international travel, and societal urbanization that produced more abundant mosquito breeding sites (e.g., refuse, discarded tires, septic tanks) [9].

**Fig. 1:**
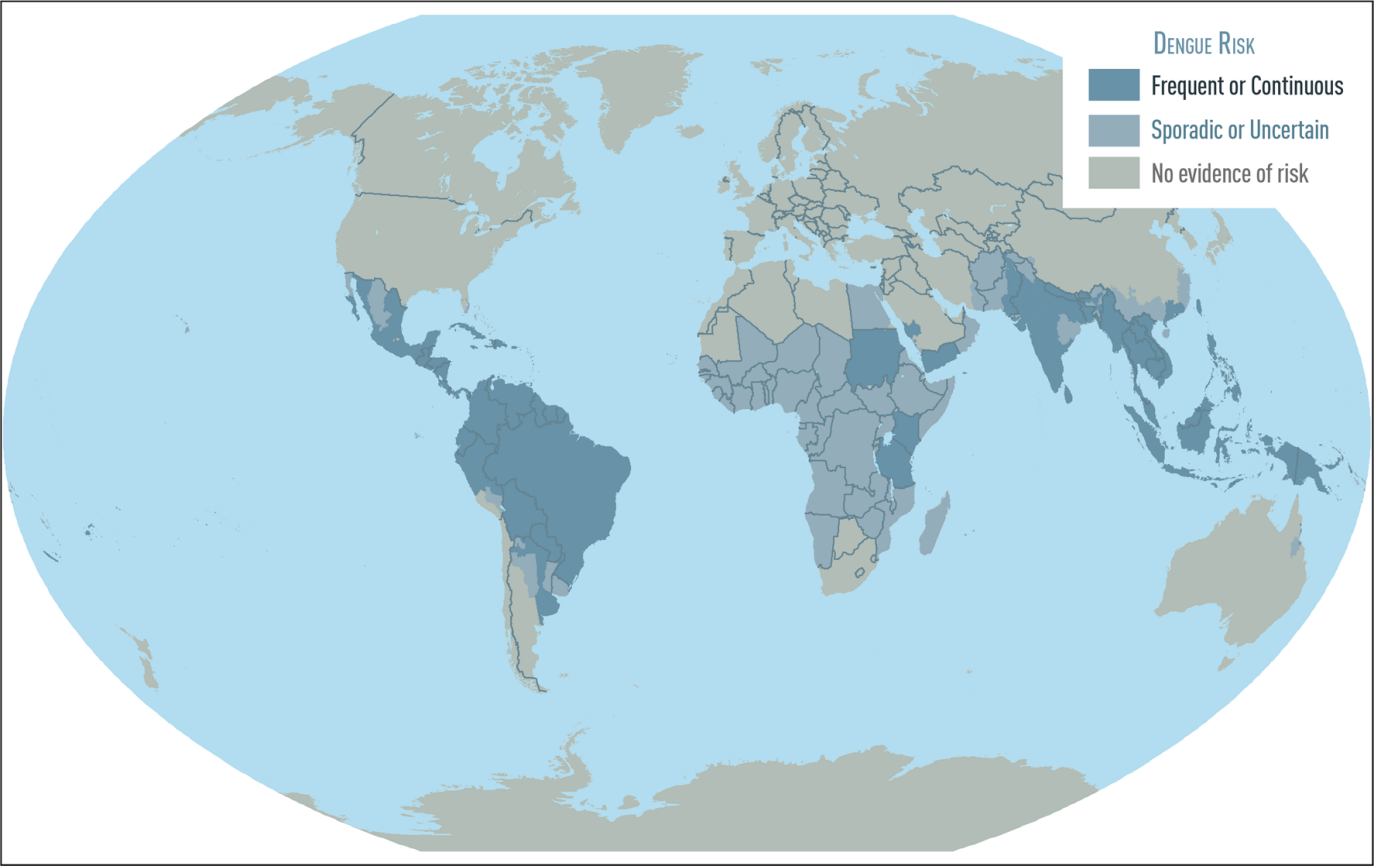
Regions of the world where there is available evidence for risk of dengue virus infection. Map provided by CDC Travelers’ Health Branch. For up-to-date information on dengue activity, please see dengue map (www.healthmap.org/dengue)

The four DENV types and their mosquito vectors are now present throughout the tropics and sub-tropics worldwide (Fig. 1) [10, 11]. Recent dengue incidence has increased greatly [8], having roughly doubled each decade since 1990 [12]. Though both annual incidence and methods utilized to estimate incidence vary, recent estimates have suggested that 96 million dengue cases occurred in 2010 [13] and 58 million dengue cases and over 9000 deaths occurred in 2013 [14•]. Longitudinal analysis of surveillance data from Southeast Asia [15] and the Americas [8] demonstrated longer and more frequent dengue epidemics and increasing dengue-related morbidity and mortality. By all measures, the worldwide economic and health-associated burden of dengue has increased dramatically since World War II, and this trend will likely continue until an effective dengue vaccine and/or sustainable control strategy is identified to prevent DENV transmission [16].

As such prevention and control strategies are implemented, measuring their effect will be critical to demonstrate efficacy in preventing disease and saving lives. Findings from evaluations can be used to develop recommendations regarding best practices in controlling dengue. Doing so will require a comprehensive understanding of the epidemiology of dengue, including the populations most affected, spectrum of clinical disease, and temporal and spatial patterns of illness. Despite several decades of study, many misperceptions regarding the epidemiology of dengue persist among the public, clinicians, and public health professionals. These misperceptions have occurred in part because much of our early knowledge of dengue was gained during the first DHF epidemics in Southeast Asia. Understanding of these epidemics was limited by a lack of population-based case identification and/or laboratory diagnostics to distinguish dengue from other causes of acute febrile illness (AFI). Specifically, many early studies of dengue in Southeast Asia were performed solely among hospitalized children suspected to have dengue [17]. As a result, dengue hemorrhagic fever (and hence, by proxy, dengue) was perceived to be an illness exclusively of childhood. Still other studies were conducted in dengue-naïve adults (i.e., soldiers) sent overseas to Asia [18]. Consequently, our initial understanding of dengue came from populations that were not representative of the entire population living in areas with endemic dengue.

This review calls attention to new findings on the epidemiology of dengue worldwide with respect to previous perceptions. In particular, these new findings include improved recognition of the spectrum of disease caused by DENV infection, particularly in adults, the mechanisms of DENV dissemination within populations, and the role of homo- and heterotypic anti-DENV antibodies in affecting DENV transmission in populations. We discuss whether these findings are due to increased clinical awareness, improved surveillance and diagnostics, demographic changes, or actual epidemiological shifts in affected populations. We provide an epidemiologic framework hopefully useful to further improve our understanding of dengue epidemiology, and we detail the additional steps needed to gain a more complete understanding of the epidemiology of dengue worldwide.

### Improved Recognition of DENV Infection as a Cause of Acute Febrile Illness

For decades, dengue has been perceived as a clinical syndrome defined by fever, body aches, leukopenia, and other symptoms of acute viral infection [19, 20]. Also recognized for several decades is the potentially fatal DHF/dengue shock syndrome associated with bleeding manifestations, capillary leakage, and elevated hematocrit. However, DENV infection can result in a variety of outcomes ranging from asymptomatic infection, to mild or sub-clinical illness, to hospitalization and death with other clinical presentations. Reclassification of dengue case definitions in the late 2000s [16] broadened the spectrum of severe dengue to improve both the clinical recognition of and surveillance for severe illness attributable to DENV infection [21]. Nonetheless, dengue-related deaths are still underrecognized in endemic areas due to reliance on passive case reporting and/or review of death certificates to identify fatal dengue cases, which may not be an accurate means by which to capture dengue deaths due to frequent misdiagnosis [12, 22–25].

On the opposite end of the clinical spectrum but far more common than severe or fatal dengue, many mild illnesses caused by DENV infection that do not conform to existing dengue case definitions are neither diagnosed clinically nor reported. Pediatric AFI surveillance in Ratchaburi, Thailand, demonstrated that more than half of patients with symptomatic DENV infection had an undifferentiated fever that did not meet the WHO dengue case definition [26, 27]. Similar observations regarding the lack of sensitivity of the WHO dengue case definition in capturing individuals with symptomatic DENV infection were made during active surveillance for febrile illness in rural Thailand [28]. These findings are not specific to Southeast Asia. In Nicaragua, one quarter of pediatric patients with febrile illness had laboratory-confirmed DENV infection but did not meet the WHO dengue case definition [29]. Because these children were enrolled in a prospective cohort study that assessed for both DENV infection and dengue, these events in many cases would not otherwise have been identified since asymptomatic and sub-clinical infections would not have come to the attention of the health care system. Thus, even in endemic areas where dengue awareness is high in both the public and clinical communities, DENV infection is underrecognized as a cause of mild illness, thereby contributing to underestimation of the global burden of illness caused by DENV infection.

The recognition of DENV infection as a cause of mild illness has in part been made possible through improved molecular [30, 31] and serologic [32] diagnostic methods that together enable accurate diagnosis with a single serum specimen. Of similar utility, particularly in areas without laboratories capable of performing RT-PCR and ELISA, is the recent availability of dengue rapid diagnostic tests (RDT) [32]. Consequently, dengue has now been detected in nearly all regions of the tropics and sub-tropics (Fig. 1), including countries thought to have endemic dengue but where illness is still unrecognized [33]. For example, dengue has long been neglected in sub-Saharan Africa [34–37], and in the southern USA where dengue is reemerging [38–40].

A necessary first step to better identify populations most affected by dengue worldwide is improving clinical awareness of dengue in the context of other etiologies of AFI. Many tropical AFIs (e.g., chikungunya, Zika, leptospirosis, influenza, scrub typhus, typhoid fever, bacterial sepsis, West Nile virus infection, acute HIV infection) share symptoms with dengue which complicates diagnosis. This challenge was demonstrated during a dengue outbreak in 2011 among African Union Mission peacekeeping troops in Mogadishu, Somalia, initially diagnosed as an outbreak of “hemorrhagic malaria” (CDC, unpublished data). Similar examples of underecognition of dengue in sub-Saharan Africa have recently come to light [36, 41–43]. Even in areas where dengue has been endemic for decades and burden of illness is well-recognized, deficiencies persist in clinical diagnosis and management of dengue patients [44, 45•]. Despite recent inroads, including use of rapid diagnostic tests to identify dengue outbreaks [46], improving the awareness and diagnosis of dengue in Africa and other areas of the tropics where dengue is neglected or underrecognized remains difficult due to low clinical suspicion of dengue as a cause of non-hemorrhagic AFI.

The consequence of underrecognition of DENV infection as a cause of mild illness is underestimation of the economic and health-associated burden of dengue worldwide [47]. Underreporting is an inherent facet of all passive surveillance systems, which are used for national reporting of dengue cases in all countries worldwide that capture such information. Enhanced surveillance in Thailand [48], Nicaragua [49], and Puerto Rico [50], all of which are countries where dengue has been endemic for decades and clinical awareness is high, has estimated underreporting of dengue through national surveillance to be between 8- and 28-fold. Underreporting of dengue in countries with non-endemic travel-associated dengue is likely to be higher than in endemic areas due to low clinical awareness, lack of availability of diagnostics, and/or inefficient infrastructure for surveillance to operate. For example, roughly 80% of >5800 laboratory-positive dengue cases in the USA were not reported during 2008–2011(CDC, unpublished data). A further consequence of underrecognition is delayed or ineffective control responses. In Thailand, vector control activities that are triggered by reported dengue cases overlooked more than half of individuals with symptomatic DENV infection since the cases did not meet reporting requirements [26]. Therefore, timely initiation of population-level interventions to control or limit DENV transmission relies on the following: (1) clinical recognition of dengue as a potential cause of the patient’s illness, (2) availability of timely and accurate laboratory diagnostics to differentiate DENV infection from other AFIs, and (3) appropriate case definitions and surveillance mechanisms to enable national case reporting.

### Dengue in Adults

The traditional view in Southeast Asia is that dengue is a pediatric illness that rarely affects adults if at all [17]. Because much of our understanding of dengue pathogenesis originated in the Southeast Asia outbreaks of DHF, this view has pervaded the perception of dengue worldwide. Consequently, most surveillance for dengue in Southeast Asia was conducted primarily in pediatric hospitals where DHF cases are predominantly reported [17]. For example, in Cambodia, only hospitalized persons aged <15 years were reported, a trend that persists in the present day [48]. Although the burden of dengue in Southeast Asia is unarguably highest in children and adolescents, contemporaneous study of dengue in adults in Indonesia demonstrated a larger burden of disease than previously realized [51]. Similarly, enhanced surveillance for dengue in Vietnam demonstrated that adult dengue patients account for one third of all dengue-related hospital admissions [52]. Population-based dengue surveillance conducted in Brazil [53], Puerto Rico [54], Malaysia [55], and Taiwan [56] demonstrated that half or more of all reported dengue cases are in adults who experience the full spectrum of clinical illness. Although the age groups most affected by dengue and severe dengue may indeed differ between Southeast Asia and the Americas, due at least in part to differences in the relative force of infection [57, 58], lack of population-based surveillance precludes comparison of these potential differences in affected age groups. Therefore, population-based surveillance for dengue should be implemented and reporting restrictions that only allow pediatric cases to be reported as suspected dengue cases should be removed. Only through these approaches can age groups affected by dengue be accurately identified and compared between countries.

Such comparisons are necessary to better understand the outcome of DENV infection between different age groups. Infection with a DENV has long been thought to be more likely to result in clinically manifest dengue in adults than in children, and recent studies have examined this finding in more detail. Studies from Brazil [59] and Vietnam [60] have demonstrated that the risk of developing dengue after first DENV infection increases with age. Similarly, studies in Brazil [61], Puerto Rico [62], Thailand [63], and Taiwan [56] have shown that illness severity correlates with age in individuals with clinically apparent dengue; however, these observations are contrary to those seen in Singapore [64]. This discrepancy is not easily resolved, as recent clinical evaluations of potential differences in dengue manifestations between children and adults conducted in Nicaragua [65], Thailand [63, 66], and Vietnam [67] have demonstrated that children more frequently develop vascular leakage and shock whereas adults have a propensity to experience hemorrhage and organ involvement. Although tempting to conclude that age-dependent differences in the response to DENV infection are responsible for differential disease manifestations between children and adults, the possibility of other explanations such as adults having underlying co-morbidities, co-infections, delayed presentation for care, and/or delayed recognition of dengue by health care providers [22, 23, 64, 68] cannot be excluded as possible explanations for these observed differences. Further investigation is therefore needed to determine if adults more frequently experience severe illness following DENV infection and/or illness onset, or if the manifestations of dengue and severe dengue are simply disparate in children and adults.

Recent findings from Thailand have demonstrated that the median age of reported dengue case patients has increased over the past decade [69]. Although several explanations for this phenomenon have been proposed, including decreased vector abundance [70], a more plausible explanation is that a demographic shift in the age structure of Thailand due to a decreasing birth rate and increasing immigration led to a decreased force of infection and a consequent increase in age of DHF cases [71]. Interestingly, these changes occurred in the context of a decreasing force of DENV transmission while the basic reproductive number was unchanged, further suggesting that the expanding age groups affected by dengue was due more to underlying changes in the demographics of the population than with changes in the dynamics of DENV transmission [58]. Should similar observations of increasing age of dengue patients be made in other countries, further elucidation of the mechanisms responsible will be needed to determine if this age shift is due to demographic or other changes.

### Neurologic Manifestions of DENV Infection

A number of investigators have documented that DENVs, while apparently less neurotropic than other flaviviruses such as Japanese encephalitis virus, West Nile virus, St. Louis encephalitis virus, and now Zika virus, cause a relatively small but significant burden of neurologic illness [72]. Neurologic manifestations include encephalopathy, encephalitis, and neuromuscular abnormalities. The incidence of dengue with neurologic complications and the proportion of febrile neurologic illness due to DENV infection varies markedly (4–47%) in reports from different settings [73]. Involvement of the central nervous system is now a criterion for severe dengue; however, standardized diagnostic criteria for neurologic manifestations of dengue are lacking [74]. Again, increased clinical awareness and systematic testing for dengue in patients with acute febrile neurologic illness using standardized clinical definitions are necessary for a clearer understanding of disease burden.

### Mechanisms of DENV Dissemination

Dengue epidemics typically occur every 3–5 years in endemic areas, and peak dengue incidence typically occurs in association with the rainy season during both epidemic and non-epidemic years [16]. Although several studies have associated the occurrence of dengue epidemics with El Niño southern oscillation (ENSO), ENSO does not independently determine the occurrence of epidemics [75]. The identification of accurate predictors of when epidemics will occur [76] has been complicated by the integral role played by mosquito populations in DENV spread; the existence of four antigenically distinct DENV types, all of which have different dynamics of infection and replication within mosquitoes and humans; and the relative contributions of homo- and heterotypic DENV immunity to herd immunity.

An established risk factor for DENV infection is sharing living space with a person with dengue [77–79], and as such dengue cases typically cluster at the household and neighborhood levels in both urban [80, 81] and rural [82, 83] settings. Mapping the travel patterns of dengue case-patients in Peru revealed that DENV transmission within communities is attributable more to mobility of infected humans than to infected mosquitoes [81], which have limited flight range and do not frequently mediate transmission outside of the household [84]. Interestingly, recent studies have revealed that a similar, but mechanistically distinct, perspective can be applied on a larger scale and may in part explain the periodicity of epidemics. Cummings and colleagues elegantly demonstrated that Bangkok, Thailand frequently serves as a dengue epicenter, from which a wave of dengue travels in humans away from Bangkok roughly every 3 years at ~148 km per month to ultimately affect nearly the entire country [85]. Similar observations implicating cities as the site of spread of epidemics have since been made in Vietnam [86, 87] and Brazil [88]. Because the introduction of new DENV clades is one factor associated with the occurrence of epidemics [89, 90] and changes in disease severity [91], a more complete understanding of whether new clades arise in cities or if cities serve to amplify new clades after they are introduced, or both, will assist in the planning of dengue control efforts.

Similar to inter-city spread of DENVs, a well-recognized mechanism of intercontinental DENV spread is via infected travelers returning from endemic areas [92]. A prospective study of more than 1200 short-term Dutch travelers to the tropics demonstrated that 14.6 DENV infections occurred per 1000 person-months of travel [93]. Similarly, risk of DENV infection was predicted to be 0.17 and 0.2% for a 1-week stay during peak dengue season in Singapore [94] and Thailand [95], respectively, which correlates well with rates observed in Israeli travelers to Thailand [96]. However, attack rates can be considerably higher during short-term travel to regions with endemic dengue among certain high risk groups. For example, 25% of a cohort of American missionaries experienced symptomatic DENV infection following a 1-week trip to Haiti during a presumptive epidemic [97]. Risk of DENV infection and likelihood of importation to travelers’ home countries varies by duration of stay, the environments encountered during travel (e.g., vacation travel to a resort versus visiting friends and family in an urban area), and intensity of DENV transmission when travel occurs (e.g., peak versus low dengue season) [98].

Hundreds of viremic travelers each year return to areas of the USA and Europe that have *Aedes* mosquitoes and a largely susceptible human population [99], demonstrating that travelers are at risk not only of becoming ill following DENV infection while abroad, but also of importing the virus and causing local outbreaks, as has recently occurred in the USA [100–102] and Europe [103]. Moreover, dengue epidemics in Taiwan [104] and China [105] have been associated with DENV importation from Southeast Asia, whereas those in Queensland, Australia, were associated with importation from Oceania [106, 107]. These findings together reinforce that travel-associated dengue cases can introduce and result in local DENV transmission, illustrating the need for improved clinical awareness and reporting of travel-associated dengue cases to implement public health measures to contain outbreaks.

### New Views on DENV Antibodies

The presence of anti-DENV antibodies that bind the virus but do not neutralize it to prevent infection of new cells is a well-established risk factor for developing severe dengue [16]. However, in relation to protecting both individuals from developing dengue and populations from experiencing epidemics, the role of DENV immunity until recently had been largely neglected. Early studies of heterotypic immunity conducted by Albert Sabin demonstrated that cross-protection against disease following infection with a DENV type different from the initial infecting type lasts several months [108]. However, contemporaneous cohort studies conducted in Nicaragua [109] and Thailand [110] estimated the duration of protective heterotypic immunity to be on the order of 1 to 3 years and demonstrated a directly proportional relationship between duration of time between infections and likelihood of developing symptomatic infection. Although the correlation of the duration of protective heterotypic antibody with the frequency of epidemics is interesting [111], the relationship between waning heterotypic antibody and its effect upon herd immunity has yet to be conclusively addressed. Finally, studies from Nicaragua and Puerto Rico have provided limited evidence for a sex-specific difference in the duration of protective heterotypic immunity [109, 112], a finding which remains to be more rigorously validated.

The same longitudinal cohort studies have shed new light on the frequency and role of homotypic DENV immunity. Though DENV infection was historically considered to result in sterilizing immunity against the infecting DENV type [108, 113], re-infection with the same DENV type has been recently documented, albeit rarely, in individuals in Nicaragua, Peru, and Puerto Rico [112, 114•, 115••]. Of interest, most homotypic DENV infections identified to date have been sequential infections with DENV-2, which was recently shown to be the most antigenically diverse of the four DENVs [2]. Since most homotypic DENV infections appear to have been either asymptomatic or subclinical, the relative contribution of homotypic DENV re-infection on burden of disease is unclear. Nonetheless, a natural history study of human DENV infections in Cambodia elegantly demonstrated that asymptomatic DENV infections do indeed lead to infection of mosquitos [116•]. Therefore, even if homotypic infections do not appreciably increase the burden of disease, they still may contribute to propagation of transmission. Although such homotypic infections have been rarely identified to date, it is indeed possible that they occur more often than has been previously appreciated but are only now being recognized due to the recent initiation of dengue cohort studies, improved case detection through enhanced or more efficient case surveillance, and/or increased use of molecular diagnostic techniques that can identify the infecting DENV type with greater reliability than serologic diagnosis [113].

Similarly, the occurrence of asymptomatic DENV infections and the role that antibodies play in the likelihood of developing symptomatic infection have recently been addressed. In Thailand, the wide yearly fluctuation between the ratio of symptomatic-to-inapparent (rS:I) DENV infections in school children in a given year is related to DENV infections in the previous year, in that the prior year’s incidence and subsequent year’s rS:I DENV infections have an inverse relationship [117]. Similar observations were made utilizing data from a prospective pediatric cohort in Nicaragua [109], where neutralizing antibody titers were observed to be a correlate of protection against symptomatic DENV infection [118•].

Although Sabin demonstrated that pre-existing antibodies against Japanese encephalitis virus (JEV), a flavivirus genetically related to DENV, protected individuals from developing dengue following DENV infection [108], in a recent school-based cohort from Thailand pre-existing anti-JEV antibody increased the likelihood of symptomatic DENV infection [119]. Conversely, the absence of clinically apparent illness due to infection with West Nile virus, also a flavivirus, despite circulation in sentinel animals in areas with endemic dengue [120, 121] suggests that anti-DENV antibodies may provide some level of protection from WNV disease. Future studies should continue to address the role of cross-reactive anti-flavivirus antibodies in the potential modulation of clinical outcome following infection with DENV or another flavivirus, including a potentially worsened clinical outcome as has been suggested by a case report of an individual who had been previously infected with a DENV and died from hemorrhagic illness following WNV infection [122].

## Conclusions

Although we now know more about dengue than ever before, much work remains before a comprehensive understanding of the modern epidemiology of dengue can be achieved. In addition, despite more than a century of research, still no effective means to prevent dengue in communities is available. Until such a solution is found, the worldwide burden of dengue is likely to continue to increase.

Despite recent advances in dengue vaccines [123, 124] and novel approaches to vector control [125], challenges in both fields [126, 127] have left optimization of patient management through increased clinical awareness as the only approach to reduce dengue-related mortality [45•]. To effectively measure the effect of interventions on the incidence and burden of dengue, as well as to consistently define the occurrence of epidemics, baseline surveillance data that are gathered equivalently throughout the world is urgently needed [128]. Moreover, implementing enhanced surveillance (i.e., sentinel health care facilities in which clinicians are trained and encouraged on how to appropriately identify, manage, and report dengue patients) in areas with existing passive surveillance systems will enable calculation of accurate rates of dengue and severe dengue, and allow for inter- and intra-country comparison of the burden due to dengue [19, 128].

A persistent limitation to understanding the burden of dengue is reporting of dengue cases to public health authorities. In the absence of consistent case reporting, an accurate understanding of the epidemiology of dengue will be difficult to achieve. An additional benefit to complementing passive surveillance with a limited number of enhanced surveillance sites is therefore to gain a more accurate estimate of the morbidity and mortality due to dengue. Implementation of enhanced surveillance sites will therefore better enable an accurate estimation of the burden of severe and fatal dengue. Furthermore, to improve case identification, availability of dengue diagnostics, both rapid tests and laboratory-based confirmatory testing, is needed to differentiate patients with dengue from other AFIs, and thus additional emphasis should be placed on improved dengue laboratory capacity worldwide, but especially in areas where dengue is neglected. Finally, without country-specific knowledge of all age groups affected by dengue, the required evidence for countries to identify the age groups that should receive a dengue vaccine is both insufficient and convoluted. Periodic population-based serosurveys can and will be useful for this purpose but are logistically challenging and labor intensive. Enhanced surveillance is the most feasible route to gain an accurate, comprehensive, and comparable understanding of the global epidemiology of dengue.
